# Reflections on the James Lind Alliance priority setting partnership in congenital heart disease

**DOI:** 10.1136/openhrt-2025-003342

**Published:** 2025-05-05

**Authors:** Nigel E Drury, Katherine L Brown, Louise Coats, Giovanni Biglino, Sarah Murray

**Affiliations:** 1 Department of Cardiovascular Sciences, University of Birmingham, Birmingham, UK; 2 Department of Paediatric Cardiac Surgery, Birmingham Children’s Hospital, Birmingham, UK; 3 Cardiac Intensive Care Unit, Great Ormond Street Hospital for Children, London, UK; 4 Institute of Cardiovascular Science, University College London, London, UK; 5 Population Health Sciences Institute, Newcastle University, Newcastle upon Tyne, UK; 6 Adult Congenital Heart Unit, Freeman Hospital, Newcastle upon Tyne, UK; 7 Bristol Heart Institute, University of Bristol, Bristol, UK; 8 National Heart and Lung Institute, Imperial College London, London, UK; 9 Society for Cardiothoracic Surgery in Great Britain and Ireland, London, UK

**Keywords:** heart defects, congenital, research design, delivery of health care

 The James Lind Alliance (JLA) is a National Institute for Health and Care Research (NIHR)-funded initiative to identify and prioritise unanswered questions for research through priority setting partnerships (PSP).^1 2^ These comprise a three-stage consultation process: an initial survey to gather uncertainties; an interim prioritisation survey and a final priority setting workshop. PSPs are considered the gold standard, providing an equitable mechanism for agreeing which future research matters most to patients, their families and clinicians, through shared decision-making. Accordingly, the NIHR has a rolling funding call across four of their research programmes for studies which address JLA PSP research priorities.^3^ Of the 180 PSPs completed to date, several have generated Top 10 lists of priorities related to cardiovascular disease in the UK, including heart surgery,^4^ advanced heart failure^5^ and cardiomyopathy.^6^ In 2021–22, we developed, conducted and reported the congenital heart disease (CHD) PSP, bringing together patients with lived experience, parents, national charities and healthcare professionals from across the UK to determine national priorities for research in children and adults with CHD.^7 8^ In this article, we reflect on our experiences, what went well, what we could have done better and what we learnt from the process, to benefit others embarking on a similar priority-setting journey.

## Engagement with the James Lind Alliance

The first step in conducting a PSP is to approach the JLA with an idea. In June 2020, following the success of the adult cardiac surgery PSP,^4^ the Society for Cardiothoracic Surgery (SCTS) Research committee identified the need for research priorities in the congenital domain, leading to two overlapping proposals from different groups: one focused solely on congenital cardiac surgery and the other encompassing all of CHD. After much debate, the latter was taken forward as it better reflected the lifelong impact of CHD on patients and their families.

## Funding

While the NIHR funds the JLA central coordinating team, it does not fund individual PSPs. With an average cost of £60 000–£80 000, external funding is therefore vital. Other cardiovascular PSPs have been funded by charity grants,^4^ institutional awards^5^ and co-funding with industry.^6^ Our PSP was funded through philanthropy, with a generous donation from George W Davies, the high street fashion entrepreneur, whose family member with CHD had undergone multiple procedures at Birmingham Children’s Hospital. The project’s appeal lay in its potential to shape the research landscape for years to come, beyond the scope of any individual study. The main costs of the PSP included staffing for two information specialists, the JLA senior advisor and an administrator (£42 000); two priority-setting workshops (£8500), reimbursement for travel expenses (£5500) and patient and public involvement (PPI) payments (£4500), according to nationally agreed rates.^9^

## Steering group

**Figure 1:**
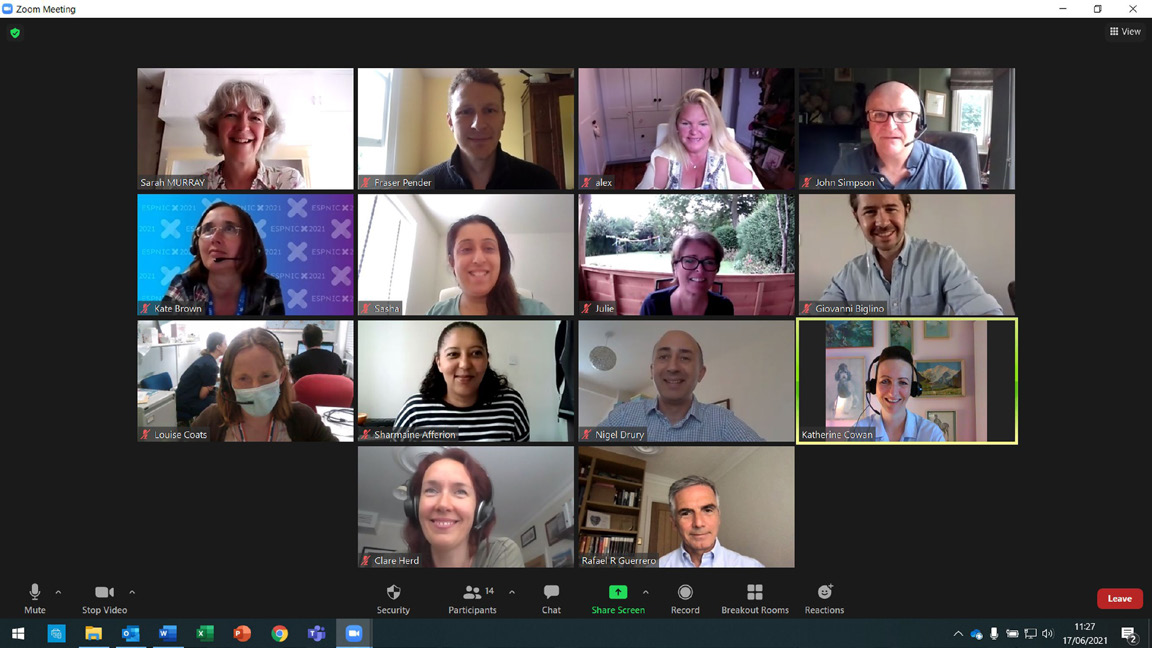
Congenital heart disease priority setting partnership steering group meeting on Zoom—14 of 17 members shown.

The JLA Guidebook offers limited advice on the composition of the steering group, other than being ‘made up of a mix of representatives of patients, carers and clinicians’ and being transparent on how they were selected.^2^ The PSP Lead invited members to join the steering group based on their research experience and expertise in different aspects of CHD, including fetal cardiology, interventional cardiology, adult congenital cardiology, congenital cardiac surgery and cardiac intensive care, from seven of the 11 UK level 1 centres, with representation of professional bodies: British Congenital Cardiac Association (BCCA) and SCTS. However, there were no nursing or allied healthcare professional representatives, which was an oversight and may have impacted on the decisions taken by the steering group. Parents of those with CHD and adults with lived experience were invited to join the committee based on their previous involvement with national charities: the British Heart Foundation (BHF), Children’s Heart Federation and Somerville Heart Foundation. As the major funder of cardiovascular research in the UK, the BHF was also invited to take part but declined due to the potential for a perceived conflict of interest. The committee was chaired by the JLA senior advisor, who brought a wealth of experience and knowledge to guide us through the process, and we met online via Zoom (Zoom Communications, San Jose, California, USA) every 4–6 weeks between March 2021 and November 2022 (figure 1). The group was gender inclusive (59% female), but mostly White British (71%) with only two members of South Asian or Black heritage.

## Scope

The CHD PSP was explicitly focused on clinical priorities for research, defined in the protocol as ‘the management of CHD throughout life, including prior to birth, focusing on diagnosis, treatment and outcomes’. The committee agreed on this approach to maximise the potential for translation into clinical trials with the potential for direct patient benefit in the next decade. However, by excluding non-management-related research domains—such as understanding the underlying causes of CHD—we may have overlooked the major translational impact that addressing these more fundamental questions could have for future generations. Despite the initial survey stating that “We are not looking at areas such as causes of congenital heart defects…”, 129 questions related to aetiology were submitted, the majority by parents. This clearly indicates that aetiology is an important concern for families. However, as it fell outside the scope of the PSP, it was not included in the final priorities. These questions will be explored further in a separate paper.

## Partners

We engaged widely with professional bodies representing key CHD stakeholders, including those in cardiology, surgery, intensive care, anaesthesia, paediatrics with a special interest, primary care, clinical perfusion and both paediatric and adult nursing. We involved UK national charities, with patient/parent representation from the Children’s Heart Federation and the Somerville Heart Foundation on the steering group, and we fostered links with other CHD-specific charities. We set up @congenitalPSP Twitter and Facebook accounts to promote the process via social media. We engaged with the BHF Heart Voices network, reaching out to Teen Heart (13–18 years) and One Beat (18–30 years) members to complete the surveys and participate in the workshops. This engagement was vital in broadening the reach of the surveys, resulting in 524 responses to the initial survey, including 133 (25.4%) patients with CHD and 247 (47.1%) parents. The child/antenatal and adult interim prioritisation surveys received 250 and 252 responses, respectively—figures that compare favourably with other UK cardiovascular PSPs.^4^,^6^ However, the number of responses to the initial survey presented a logistic challenge; over many weeks, the steering group used an iterative process to combine similar or overlapping questions, reducing the initial 1060 valid questions to a final list of 59 child/antenatal and 49 adult summary questions to proceed to the evidence-checking stage.

## Inclusivity of the process

The steering group was strongly motivated to achieve an inclusive JLA process, with a particular focus on engaging underserved communities. Inclusion of individuals from South Asian and Black backgrounds was particularly important, given that children from these backgrounds are disproportionately affected by CHD in the UK.^10^ Research shows that the incidence of severe and complex heart defects—those associated with high infant mortality—is approximately twice as high in these groups compared with the White population.^11^ In addition, the proportion unable to ‘speak English well’ is higher among those of South Asian ancestry (Asian-Indian 7.4%, Asian-Pakistani 11.2%, Asian-Bangladeshi 16.2%) than those of Black ancestry (Black-Caribbean 0.3%, Black-African 3.8%).^12^ To facilitate inclusivity, we collaborated with the Centre for Ethnic Health Research at the University of Leicester to translate the survey into Urdu, Bengali, Gujarati and Hindi—the four most commonly spoken non-English languages among South Asian communities—alongside English, Welsh and Polish, which remains the most commonly spoken immigrant language in the UK.^12 13^ Posters in five languages, featuring a quick response code linking to the online survey, were distributed via community networks and mosques. Additionally, printed surveys were handed out to targeted demographics during clinic appointments. The translated versions of the survey were made available for download and printing, and a Freepost address was set up to facilitate survey returns. The PSP Lead also appeared on ‘Living the Life’, a prime-time contemporary lifestyle show on the Islam Channel, on 2 September 2021, with the aim of promoting the survey among Muslim communities.^14^ The Islam Channel, a UK-based, free-to-air, English-language television channel, reportedly has 2 million viewers in the UK alone, reaching approximately 60% of British Muslims.^15^

Despite these efforts, only 17 (4.4%) of the 387 responses from parents, patients or other family members to the initial survey identified as having Asian heritage, and only two (0.5%) identified as Black.^7^ No respondents completed any of the translated versions, and only one response in English was returned via the Freepost address. The combined costs of translating the survey and poster, as well as setting up and administering the Freepost address, amounted to nearly £1000. Similarly, following the 20 min live television interview on the importance of CHD research to a Muslim audience, no responses were received from individuals of Asian or Black heritage in the subsequent week. Throughout the initial survey period, we actively monitored respondent demographics, and in response to the lack of ethnic diversity, we initiated targeted engagement with families during clinic appointments. This was the only strategy that appeared to increase participation from individuals of Asian or Black backgrounds.

## One process, two lists of priorities

At the outset, the steering group anticipated that parents, especially mothers, would be the most engaged group, as found in a previous national CHD prioritisation exercise.^16^ Additionally, it was noted that more healthcare specialists work with children with CHD than with adults. To ensure that priorities relevant to the growing population of adults living with CHD were not overshadowed, we agreed to split the process into parallel child/antenatal and adult tracks at the interim prioritisation stage. This approach resulted in two separate Top 10 lists and marked the first time a JLA PSP deviated from the prescribed pathway in this way, effectively undertaking two PSPs within a single process.

This approach successfully achieved its goal of ring-fencing adult-specific priorities. As expected, parents comprised the largest group of respondents to the initial survey (247; 47.1%), compared with 109 adults living with CHD (20.8%). However, adult engagement increased significantly at the second stage, with 151 adults (59.9%) responding to the adult prioritisation survey. This approach resulted in four adult-specific priorities being included in the Top 10: pregnancy (#5), heart failure—particularly in those with a systemic right ventricle (#6), arrhythmias including sudden cardiac death (#7) and transplantation and long-term mechanical support (#8). Most, if not all, of these may have been excluded from a single, paediatric-dominated Top 10 list.^7^ However, this separation may have come at the expense of issues related to transition to adult services, a significant concern for both families and clinicians, which ultimately failed to break through to the final priorities.

## Workshops

Two workshops—one focused on child/antenatal priorities and the other on adult priorities—were held on consecutive days in June 2022 at a central Birmingham venue within walking distance of a major rail hub. However, attendance at the paediatric workshop was affected by national rail industrial action, which disrupted travel. As a contingency measure, several local clinicians were on standby to cover gaps in expertise caused by last-minute absences. Despite the logistic challenges, 10 of the 12 CHD networks in the UK and Ireland were represented at one or both workshops, ensuring a broad range of interests and clinical expertise.

The two workshops were attended by independent groups of delegates, and a clear consensus emerged rapidly on both days.^8^ Remarkably, six of the priorities in each of the Top 10 lists were derived from the same summary questions, triangulating and highlighting their importance throughout life, while the four child/antenatal-specific and four adult-specific priorities provide a focused platform to address age-specific issues. Many of the priorities encompass holistic outcomes, extending beyond early mortality to improve the quality of survivorship and reduce the impact of living with CHD. This provides a strong platform for conducting research that truly matters to those who may directly benefit. However, it was also important to manage expectations—particularly among parents—that identifying key priorities marks only the beginning of a much longer journey towards improved care and outcome.

## Publication and impact

The main output from the PSP was published in *Open Heart* in December 2022, enabling rapid dissemination of our findings to a wide audience. All patient, parent and clinician members of the steering group were included as coauthors.^7^ By the end of August 2024, the full text had been accessed 5913 times and the PDF downloaded 916 times, with 15 citations in the first 2 years. These metrics suggest that our findings are already influencing the research agenda, although it is still too early to determine their impact on patients, charities or clinical care.

## Beyond the priority setting partnership

Most PSPs publish their Top 10 list and leave it to the wider community to address the priorities. However, given the scarcity of research collaborations between CHD centres in the UK and Ireland, we felt strongly that we should use the priorities as a platform to bring the community together, to develop more collaborative research for the benefit of all. This led to the publication of ‘*Transforming collaborative research: a national strategy to address the James Lind Alliance priorities for children and adults with congenital heart disease*’ in July 2023,^17^ which was endorsed by BCCA, SCTS and national charity partners, and supported by NHS England National Specialised Commissioning and the CHD Clinical Reference Group. The central objective of this strategy is the establishment of the Congenital Heart Research Network, a UK and Ireland collaborative network for multicentre studies, focusing on clinical trials and other studies that have the potential to change clinical practice and improve outcomes in congenital and paediatric inherited and acquired heart disease. This will be supported by a national PPI group, comprising engaged patients, parents, carers and charities with lived experience or affected by CHD, to actively contribute through all stages of the design, conduct and reporting of research. The development of this network was informed by the experiences of the Paediatric Heart Network in North America,^18^ the UK National Adult Cardiac Surgery Clinical Trials Initiative^19^ and the UK Paediatric Critical Care Society Study Group.^20^ The proposal was approved by the BCCA Council and SCTS Research Committee (box 1), and with support and funding from the BHF Clinical Research Collaborative and a philanthropic donation from the Miskin family, a launch workshop will be held in May 2025. The success of this venture will be determined by its ability to deliver multicentre studies, integrate participation in research into routine clinical care in CHD, develop the next generation of clinical researchers, inform international clinical guidelines and deliver better outcomes for patients and their families.

Box 1 **Congenital Heart Research Network: mission statement and objectives**

**Mission statement**

1. To improve the health outcomes and lives of those affected by congenital and paediatric inherited & acquired heart disease.
2. To support and conduct high-quality, multicentre collaborative research to drive evidence-based care.
3. To involve patients, families and charities in all aspects of the research.
4. To improve the research knowledge and skills of the congenital heart disease (CHD) workforce.

**Objectives**

1. To bring together all CHD centres in the UK and Ireland in an open, inclusive, equitable and transparent collaboration for research.
2. To provide a framework to support investigators to develop and lead multicentre studies, primarily clinical trials but including other research such as prospective observational studies, analyses of routinely collected data, systematic reviews and surveys of practice.
3. To facilitate patient and public involvement and engagement, with representation and integration through all stages of the research lifecycle.
4. To work with other national organisations to encourage better use of routinely collected data for CHD research.
5. To develop a platform of training to enhance the research knowledge and skills of the CHD workforce, including trainees to become the next generation of research leaders.

## Conclusions

In summary, the PSP brought together patients, their families and clinicians to identify and prioritise research across CHD through shared decision-making. We focused on aspects of clinical management to maximise the potential for translation into practice-changing clinical trials, at the expense of more fundamental questions such as the cause of CHD and how to prevent it. We engaged widely with stakeholders, with a reassuring number of responses to the surveys but achieved limited engagement with South Asian and Black communities despite extensive efforts. Our strategy of splitting the process into antenatal/child and adult tracks achieved its goal of protecting age-specific priorities. Finally, while the priorities provide a platform for conducting the research that matters most, the strategy aims to establish a UK and Ireland research network for collaborative research to support clinical trials and other studies that have the potential to change practice and improve outcomes. It is vital that improving engagement with underserved communities is prioritised by the network during the development and conduct of future research.^21^
